# Application of the Still-Face Paradigm in Early Screening for High-Risk Autism Spectrum Disorder in Infants and Toddlers

**DOI:** 10.3389/fped.2020.00290

**Published:** 2020-06-05

**Authors:** Nana Qiu, Chuangao Tang, Mengyao Zhai, Wanqing Huang, Jiao Weng, Chunyan Li, Xiang Xiao, Junli Fu, Lili Zhang, Ting Xiao, Hui Fang, Xiaoyan Ke

**Affiliations:** ^1^Nanjing Brain Hospital Affiliated to Nanjing Medical University, Nanjing, China; ^2^School of Biological Science & Medical Engineering, Southeast University, Nanjing, China; ^3^College of Telecommunications & Information Engineering, Nanjing University of Posts and Telecommunications, Nanjing, China; ^4^Wuxi Children's Hospital, Wuxi, China

**Keywords:** high-risk autism spectrum disorder, the Still-Face Paradigm, social behavior, machine learning, model for early screening

## Abstract

**Background:** Although autism spectrum disorder (ASD) can currently be diagnosed at the age of 2 years, age at ASD diagnosis is still 40 months or even later. In order to early screening for ASD with more objective method, behavioral videos were used in a number of studies in recent years.

**Method:** The still-face paradigm (SFP) was adopted to measure the frequency and duration of non-social smiling, protest behavior, eye contact, social smiling, and active social engagement in high-risk ASD group (HR) and typical development group (TD) (HR: *n* = 45; TD: *n* = 43). The HR group was follow-up until they were 2 years old to confirm final diagnosis. Machine learning methods were used to establish models for early screening of ASD.

**Results:** During the face-to-face interaction (FF) episode of the SFP, there were statistically significant differences in the duration and frequency of eye contact, social smiling, and active social engagement between the two groups. During the still-face (SF) episode, there were statistically significant differences in the duration and frequency of eye contact and active social engagement between the two groups. The 45 children in the HR group were reclassified into two groups after follow-up: five children in the N-ASD group who were not meet the criterion of ASD and 40 children in the ASD group. The results showed that the accuracy of Support Vector Machine (SVM) classification was 83.35% for the SF episode.

**Conclusion:** The use of the social behavior indicator of the SFP for a child with HR before 2 years old can effectively predict the clinical diagnosis of the child at the age of 2 years. The screening model constructed using SVM based on the SF episode of the SFP was the best. This also proves that the SFP has certain value in high-risk autism spectrum disorder screening. In addition, because of its convenient, it can provide a self-screening mode for use at home.

**Trial registration:** Chinese Clinical Trial Registry, ChiCTR-OPC-17011995.

## Introduction

Autism spectrum disorder (ASD) is a serious neurodevelopmental disorder that starts in early childhood and is characterized by social communication barriers, restricted interests, repetitive stereotyped behaviors, and abnormalities in perception (1). In recent years, epidemiological survey data on the incidence of ASD showed that the prevalence rate increased from 0.07 to 1.8% in China (2). A large number of studies on ASD have shown that early intervention helps improve patient prognosis (3). However, age at ASD diagnosis is still 40 months or even later (4). Therefore, early detection, early diagnosis and effective intervention are essential to achieve a better prognosis. Understanding the early childhood behaviors of ASD will help facilitate early intervention in infants and toddlers who are suspected of having ASD and improve their prognosis, which has very important social and economic implications.

The early social interaction between adults and children is the basis of more complex social cognition. For children with ASD, the lack of social abilities is especially prominent in their early life. According to the 2013 version of the *Diagnostic and Statistical Manual of Mental Disorders*, fifth edition (*DSM-5*), ASD symptoms are usually present in young children at the age of 1–2 years. Occasionally, initial symptoms often involve delayed language development, accompanied by a lack of social interest or unusual social interactions, quirky play modes and unusual communication patterns (1). A study by Barbaro and Dissanayake on family videos and parental reports revealed early warning signs in social interactions of children with ASD aged 12 to 24 months old (5), which included lack of joint attention, lack of eye contact, lack of social smiling, lack of social interest and sharing, no response to calling name, lack of gestures, and communication impairments (5–9). This study would focus on the differences of early social behavior between HR group and TD group. Previous studies on early behavioral abnormalities in ASD were mostly in the form of retrospective interviews with parents or scale-based evaluations, which are highly subjective and unfavorable for widespread promotion. In recent years, an increasing number of studies have adopted objective methods involving video for using behavioral coding (10–12).

Tronick et al. proposed the still-face paradigm (SFP) to test infants' emotion regulation ability and social expectations in social interaction (13). Early maternal-infant interaction is the core basis of infants' social emotion, emotion regulation, and social and communication development (14). The maternal-infant relationship is the first relationship developed in early childhood. Flexible and frequent interaction is the basis for early childhood emotional organization, attention switching and the emergence of social skills (15). In the early maternal-infant interaction, non-verbal communication is dominant. In addition, in this process, young children learn the rules of social participation and expressing forms of social expectations, which provide a social framework for future social interactions and relationships (16). There were studies applied SFP in emotional regulatory of ASD and they found most of children with ASD employed more simple regulatory behavior and less complex strategies (17, 18). Additional, Cassel et al. also found that there were difficulties for children with ASD to develop socioemotional ability (10, 19). In this study, the SFP was used to measure social behavior of HR group and TD group.

There were studies applied machine learning methods to build model for early screening of ASD based on the characteristic values of biological indicators, such as electro-encephalogram (20) and brain images (21), and they found the accuracy was more than 80%. And in order to improve the stability and reliability of model for early screening of ASD, it is essential to combining social behavioral indicators with biological indicators in the subsequent research. In this study, we would try to build a model for early screening of ASD based on social behavioral indicators.

## Methods

### Participants

Forty-five infants and toddlers with high-risk autism spectrum disorder (HR) who sought treatment at the outpatient clinic of Child Mental Health Research Center, Nanjing Brain Hospital Affiliated to Nanjing Medical University, from December 2017 to December 2018 were enrolled in the HR group, and 43 infants and toddlers with typical development (TD) in the Nanjing area were recruited during the same period and enrolled into the control group (the TD group).

The inclusion criteria for the HR group were as follows: (1) children with positive results based on the Modified Checklist for Autism in Toddlers (M-CHAT) (22); (2) pediatric psychiatrist recognized that the children met the core criteria of ASD in DSM-5 but the months age was under 24 months; (3) children aged 8 to 23 months old; (4) children whose primary caregiver was the mother; and (5) children whose guardian(s) agreed to participate in this study. The exclusion criteria for the HR group were as follows: (1) children with genetic or metabolic diseases, such as Rett syndrome and fragile X syndrome; (2) children with neurodevelopmental disorders other than ASD, such as language development disorders alone and intellectual disability; (3) children with a clear history of craniocerebral trauma; and (4) children with a history of nervous system diseases and serious physical illnesses.

The inclusion criteria for the TD group were as follows: (1) children with TD whose sex matched that of the children in the HR group; (2) children aged 8 to 23 months old; (3) children whose primary caregiver was the mother; and (4) children whose guardian(s) agreed to participate in this study. The exclusion criteria for the TD group were as follows: (1) children who suffered from various types of neurodevelopmental disorders and mental disorders; (2) children with a clear history of craniocerebral trauma; and (3) children with a history of nervous system diseases and serious physical illness.

This study was approved by the Medical Ethics Committee of Nanjing Brain Hospital Affiliated to Nanjing Medical University (2017-KY089-01). All subjects' guardians agreed to participate in this study and signed informed consent forms.

### Measure and Procedure

#### General Psychological Evaluation of the HR and TD Groups

A self-guided general information questionnaire was used to collect the general demographic data, past history, medication history and family history of the study participants.

The Gesell Developmental Scale was used to assess the developmental levels of all subjects after they were enrolled. The Gesell Developmental Scale was used to evaluate the developmental quotient (DQ) of children from 5 skill domains: adaptive, gross motor, fine motor, language and personal-social.

The severity of ASD symptoms in the HR group was assessed using the Communication and Symbolic Behavior Scales Developmental Profile (CSBS-DP), the Childhood Autism Rating Scale (CARS) and the Autism Behavior Checklist (ABC). The CSBS-DP has 3 factor scores (social communication, language, and symbolic behavior) and a total score. The lower the CSBS-DP factor scores are, the more serious the ASD symptoms. The CARS and the ABC only have a total score. The higher the total scores are, the more severe the ASD symptoms.

#### Video of the Behaviors of the HR and TD Groups in the SFP

The classic paradigm consists of three episodes: (1) the baseline episode (face-to-face interaction, FF episode), during which the mother and the child are required to have normal interactions; (2) the still-face (SF) episode, during which the mother's face is required to present a neutral expression without any response to the child's action; and (3) the reunion episode, during which the mother resumes normal interactions with the child. Currently, the SFP can arouse children's behavioral changes, e.g., a reduction in eye gaze and positive facial emotion and an increase in negative emotion when transitioning from the baseline episode to the SF episode (13); this effect has been recognized and termed the SF effect. In relevant studies, researchers also found that the reason for the generation of the SF effect in infants and toddlers is the disappearance of social responses, such as eye contact, when their mothers show a still face; the disappearance of social signals causes the appearance of negative emotions in infants and toddlers (23). The first two episodes are often used in research in a randomly presented order. In the past few decades, the basic settings of the SFP have been used as a method to explore early childhood social behaviors (24–26), which the reason why this study chosen the first two episodes.

**Figure 1:**
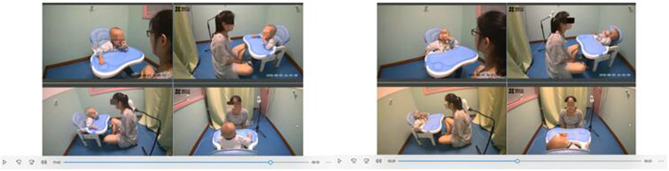
SFP for infants and toddlers with HR.

In the present study, all participants and their mothers were video recorded during the SFP in a designated observation room at the time of enrollment. The mother was sitting opposite to the child, interacted with the child for 2 min under fixed instructions, and then stopped the interaction and maintained a neutral face for 1 min. Figure 1 shows the setup of the experimental environment and procedure.

Infant and Caregiver Engagement Phases (ICEP) (27) and Nichols et al. (28) define the coding indicators as follows: (1) protest behavior - the infant shows facial expressions of anger and frowns; the infant is upset, crying, arching the body, trying to escape and expresses anger using gestures; (2) non-social smiling—the infant smiles not at the mother but toward other directions or at other objects; (3) eye contact—the infant looks directly at the mother's eyes or face instead of looking at the camera or toward other directions; (4) social smiling—the infant looks at the mother and takes the initiative to smile (initiating a smile); after the mother smiles, the infant immediately responds with a smile (smiling at each other); and (5) active social engagement—the infant displays happy facial expressions, including a clear smile, occasional cooing or active vocalization, laughing, or babbling, and looks at the mother to initiate interactions (proactively initiate active social engagement), or the infant has a positive response to the interaction initiated by the mother (responding to active social engagement).

The Observer XT 12 behavioral observation and recording analysis system was used for coding. Video coding was completed by 2 trained graduate students, and the duration and frequency of the 5 indicators during the FF and SF episodes were calculated. The duration was measured using seconds (s) as the unit, and frequency was measured using the number of times as the unit. A total of 18 videos (~20%) were randomly selected to determine intercoder consistency using the intraclass correlation coefficient (ICC). It was found that the 2 coders had high consistency: the ICCs for protest behavior, non-social smiling, eye contact, social smiling, and active social engagement were 0.76, 0.82, 0.81, 0.83, and 0.79, respectively.

#### Diagnostic Evaluation of Children in the HR Group at 2 Years of Age

At 2 years of age, the children in the HR group were evaluated using the Autism Diagnosis Interview-R (ADI-R) and the Autism Diagnostic Observation Schedule (ADOS). Two pediatric psychiatrists then clinically diagnosed the children based on the ASD diagnostic criteria in the DSM-5 and the aforementioned evaluation results. All participants with confirmed ASD (the ASD group) reached the cut-off scores for ASD diagnosis for both evaluation scales.

#### Analytic Approach

The sex difference between the HR and TD groups was compared using the χ^2^ test. The differences between the HR group and the TD group in social behaviors were determined using the independent samples *t-*test. The correlations of social behaviors in the HR group with age, DQ, and symptom severity were analyzed using Pearson's rho. Finally, models for early ASD screening were constructed using machine learning methods based on ASD group and TD group, and the HR group (contained 40 children with confirmed ASD and 5 children with not met the criterions of ASD) would be used to verified the effectiveness of models for early ASD screening. *P* < 0.05 indicated that the difference was statistically significant.

## Results

### Comparison of the General Conditions Between the HR Group and the TD Group

Age (months), adaptive DQ, language DQ, fine motor DQ, and personal-social DQ were significantly different (*P* < 0.05) between the HR group and the TD group, while sex DQ and gross motor DQ were not significantly different (*P* > 0.05) between the two groups. See Table 1 and Figure S1.

**Table 1:** Comparison of the general conditions between the HR group and the TD group (*mean* ± *SD*).

		**HR group**	**TD group**	***t*/**χ^2^****	***P-*value**
		**(*n* = 45)**	**(*n* = 43)**		
**Sex**				−0.06	0.08
	Male	40	32		
	Female	5	11		
**Age (months)** **DQ**		19.71 ± 3.43	16.40 ± 4.70	3.80	<0.01
	Adaptive	80.29 ± 17.62	92.98 ± 7.89	−4.34	<0.01
	Gross motor	92.02 ± 17.60	92.77 ± 8.46	−0.25	0.80
	Fine motor	86.64 ± 19.03	93.70 ± 8.29	−2.24	0.03
	Language	60.84 ± 21.27	86.51 ± 8.353	−7.39	<0.01
	Personal-social	78.80 ± 17.19	92.28 ± 7.18	−4.76	<0.01

*HR, high-risk autism spectrum disorder; TD, typical development; DQ, developmental quotient of the Gesell Developmental Scale*.

### Comparison of the Social Behaviors Between the HR and TD Groups During Different SFP Episodes

**Figure 2:**
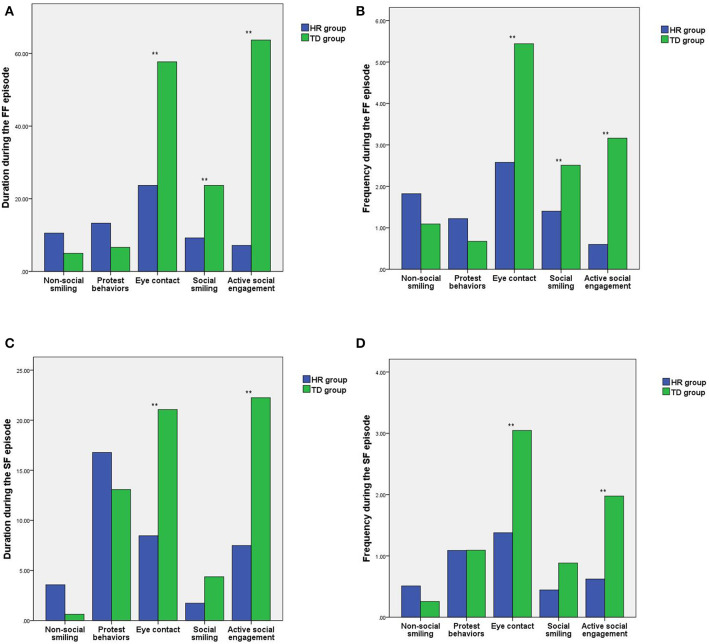
Comparison of the differences in social behaviors between the HR group and the TD group during the different SFP episodes. **(A)** Comparison of the differences in the duration of social behaviors during the FF episode. **(B)** Comparison of the differences in the frequency of social behaviors during the FF episode. **(C)** Comparison of the differences in the duration of social behavior during the SF episode. **(D)** Comparison of the differences in the frequency of social behaviors during the SF episode. HR, high-risk autism spectrum disorder; TD, typical development; SFP, still-face paradigm; **P < 0.01.

During the FF episode of the SFP, there were statistically significant differences in the duration and frequency of eye contact, social smiling, and active social engagement between the HR group and the TD group (*t* =-4.93, −6.17, −3.54, −2.90, −9.56, −8.34; all *P* < 0.05), while the differences in length and frequency of non-social smiling and protest behaviors between the HR group and the TD group were not statistically significant (*t* = 1.89, 1.69, 1.62, 1.55; all *P* > 0.05). See Figures 2A,B.

During the SF episode of the SFP, there were statistically significant differences in the duration and frequency of eye contact and active social engagement between the HR group and the TD group (*t* = −4.94, −5.34, −4.49, −6.16; all *P* < 0.05), while the differences in length and frequency of non-social smiling, protest behaviors and social smiling between the HR group and the TD group were not statistically significant (*t* = 1.91, 1.33, 0.80, −0.01, −1.98, −1.71; all *P* > 0.05). See Figures 2C,D.

### Analysis of the Correlation of Social Behaviors With Each Factor for the HR and TD Groups During the Different SFP Episodes

In the TD group, the frequency of eye contact was significantly positively correlated with gross motor DQ for the SF episode (*P* < 0.05); the other indicators had no statistically significant correlations with age and DQs (*P* > 0.05; Table 2).

**Table 2:** Analysis of the correlation of social behavior with age and DQ in the TD group during different SFP episodes (*r-*value).

**Episodes and indicators**	**Age** **(months)**	**Adaptive DQ**	**Gross motor DQ**	**Fine motor DQ**	**Language DQ**	**Personal-social DQ**
**Duration during the FF episode (s)**						
Eye contact	−0.17	0.20	0.09	0.09	0.15	0.09
Social smiling	−0.04	−0.06	−0.05	−0.22	−0.04	−0.11
Active social engagement	−0.03	−0.18	−0.12	−0.25	0.11	−0.12
**Frequency during the FF episode (number of times)**						
Eye contact	−0.04	0.09	−0.10	0.11	0.03	0.01
Social smiling	0.15	−0.07	−0.08	−0.21	0.03	−0.09
Active social engagement	0.05	−0.15	−0.04	0.02	0.02	−0.14
**Duration during the SF episode (s)**						
Eye contact	0.05	−0.09	−0.02	−0.02	0.04	−0.12
Social smiling	0.04	−0.02	0.19	0.06	0.07	0.12
Active social engagement	−0.13	−0.17	0.07	−0.15	0.13	−0.01
**Frequency during the SF episode (number of times)**						
Eye contact	0.01	−0.08	0.31^*^	0.01	0.04	−0.22
Active social engagement	−0.12	−0.03	0.02	−0.04	0.14	−0.01

TD, typical development; SFP, still-face paradigm; DQ, developmental quotient of the Gesell Developmental Scale;

* *P < 0.05*.

During the FF episode, the duration of eye contact, social smiling, and active social engagement for the HR group were significantly positively correlated with the adaptive DQ, and the duration of eye contact and the gross motor DQ were also significantly positively correlated. During the SF episode, the duration of eye contact was significantly positively correlated with the language DQ and the fine motor DQ, and the duration of active social engagement and the language DQ were also significantly positively correlated (all *P* < 0.05). The other indicators had no significant correlation with age and DQs (*P* > 0.05; Table 3).

**Table 3:** Analysis of the correlation between social behaviors and age (months), DQ, and clinical symptoms of the HR group during different SFP episodes (*r-*value).

**Episodes and indicators**	**Age** **(months)**	**DQ**					**CSBS-DP**				**CARS**	**ABC**
		**Adaptive**	**Gross motor**	**Fine motor**	**Language**	**Personal-social**	**Social communication factor**	**Language factor**	**Symbolic behavior factor**	**Total score**		
**Duration during the FF episode (s)**												
Eye contact	−0.08	0.43^**^	0.31^*^	0.18	0.25	0.29	0.09	−0.01	−0.01	0.03	0.07	−0.15
Social smiling	−0.11	0.36^*^	0.25	0.08	0.21	0.14	0.10	−0.01	−0.04	0.02	0.01	−0.12
Active social engagement	−0.04	0.39^*^	0.26	0.18	0.16	0.20	0.27	−0.01	0.19	0.19	−0.04	−0.08
**Frequency during the FF episode (number of times)**												
Eye contact	−0.18	0.23	0.21	0.28	0.10	0.09	0.14	−0.04	0.05	0.05	−0.13	−0.24
Social smiling	−0.09	0.09	0.11	0.14	0.01	0.17	0.01	−0.11	−0.02	−0.06	−0.04	−0.13
Active social engagement	−0.05	0.17	0.03	0.22	−0.07	0.03	0.21	−0.07	0.21	−0.10	0.15	0.11
**Duration during the SF episode (s)**												
Eye contact	−0.04	0.29	0.22	0.37^*^	0.30^*^	0.18	0.27	0.17	0.32^*^	0.30^*^	−0.34	−0.25
Active social engagement	0.02	0.27	0.23	0.26	0.38^*^	0.12	0.29	0.24	0.29	0.30^*^	−0.11	−0.16
**Frequency during the SF episode (number of times)**												
Eye contact	−0.29	0.17	0.12	0.29	0.26	0.12	0.35^*^	0.33^*^	0.25	0.36^*^	−0.22	−0.18
Active social engagement	−0.18	0.15	0.27	0.29	0.28	0.29	0.15	−0.25	0.15	−0.18	0.13	−0.17

HR, high-risk autism spectrum disorder; SFP, still-face paradigm; DQ, developmental quotient of the Gesell Developmental Scale; CSBS-DP, Communication and Symbolic Behavior Scales Developmental Profile; CARS, Childhood Autism Rating Scale; ABC, Autism Behavior Checklist;

* P < 0.05,

** *P < 0.01*.

The analysis of the correlation between clinical ASD symptom severity and social behavior indicators suggested that there was no statistically significant difference between social behavior indicators and clinical symptom severity during the FF episode (*P* > 0.05); during the SF episode, the duration of eye contact was positively correlated with symbolic behavior factor score and the total CSBS-DP score, the duration of active social engagement was positively correlated with the total CSBS-DP score, and the frequency of eye contact was positively correlated with the social communication factor score, the language factor score, and the total CSBS-DP score (all *P* < 0.05). The other indicators had no significant correlation with the symptom severity (*P* > 0.05; Table 3).

### Using Machine Learning to Construct Models for Early ASD Screening

Through the follow-up of the HR group and the re-diagnosis of the HR group at 2 years of age, we found that 5 (1 female and 4 males) out of the 45 infants and toddlers with HR no longer met the diagnostic criteria for ASD [the non-ASD (N-ASD) group]; the other 40 children still met the diagnostic standard (the ASD group). And then the models for early ASD screening were constructed using machine learning methods based on ASD group and TD group.

We used the duration and frequency of eye contact, active social engagement, and social smiling during the FF episode as well as the duration and frequency of eye contact and active social engagement during SF episode as the behavioral characteristics of the samples. The ASD group and the TD group were used as the samples. In the classification of ASD (40 samples) and its comparison group of TD (43 samples), each subject within these 83 samples was used for testing the model that was trained on the rest 82 samples. Then, the prediction labels of each test sample corresponding to 83 classification models were collected to calculate the overall accuracy on this dataset. There were 82 training samples, and 1 sample was selected for testing. The test set data were classified using the following machine learning methods: support vector machine (SVM), naïve Bayes and random forest. And the Python platform was used for analyses. The Random Forest Classifier, Gaussian NB and SVM functions in Scikit-learn toolbox developed by Python were employed for building classification models, respectively.

The results showed that the accuracy of Bayesian classification was 80.54% for the FF episode and 82.35% for the SF episode, and that the accuracy of random forest classification was 80.72% for the FF episode and 83.13% for the SF episode. And the accuracy of SVM classification was 81.18% for FF episode and 83.35% for the SF episode, which has higher accuracy. The confusion matrix was showed in Table 4. And in order to find the age differences between the kids who were not picked up by the machine learning, ASD group and TD group (total 83 kids) was divided into 4 groups with month age, respectively, 8–11, 12–15, 16–19, and 20–23 (Figure S2).

**Table 4:** The confusion matrix of SVM.

	**ASD**	**TD**
**FF episode**		
ASD	35	5
TD	10	33
**SF episode**		
ASD	34	6
TD	7	36

*ASD, autism spectrum disorder; TD, typical development; FF episode, face to face interaction; SF episode, still-face episode*.

Subsequently, the effectiveness of the SVM classification model was verified in 40 children with confirmed ASD and 5 N-ASD children in the HR group. Unfortunately, even though the average classification accuracy of SVM was more than 80%, the 5 N-ASD was not classified correctly. The lack of enough samples of N-ASD resulted in a large imbalance between two groups. Such limited samples with only 10 indicators within each sample could be a possible reason for low recall rate of N-ASD samples. Therefore, it is essential to expand the sample of N-ASD in future study to verify the effectiveness of the SVM classification model. What's more, it is necessary to build more effective machine learning model in following study.

## Discussion

Face-to-face interaction constitutes the beginning of early childhood learning and defining social interaction, and face-to-face interactions between infants and toddlers and primary caregivers allows the former to learn (1) the meaning of self-expression behaviors; (2) the characteristics of people with whom they have a close relationship; and (3) emotional information and the perception of local culture, primary caretaker identity and self-identity (13). Emotion regulation is an important link of early childhood development milestones and is closely related to primary caregivers (29). Studies have shown that strong emotion regulation abilities in children is associated with good development and can predict social emotional outcomes at later stages (30–32) and that weak emotion regulation abilities during early childhood are associated with behavioral problems and development problems at later stages (29, 33, 34). Especially from 4 to 9 months old, infants quickly learn how to regulate emotions through face-to-face interactions; therefore, the quality of infant-mother interactions is crucial at this stage (24, 35).

Through comparative analysis of the differences in social behaviors between infants and toddlers with HR and infants and toddlers with TD before the age of 2, compared with infants and toddlers with TD, infants and toddlers with HR exhibited shorter durations and lower frequencies of eye contact, social smiling, and active social engagement during the FF episode of the SFP. This finding is consistent with those of most studies (28, 36–38). In the SF episode, compared with infants and toddlers with TD, infants and toddlers with HR showed shorter durations and lower frequencies of eye contact and active social engagement, which means that although children with HR exhibited behaviors to attract the attention of the non-responsive mothers, their ability to initiate active social engagement was lower than that of children with TD. For infants with HR, avoiding eye contact results in a low-quality infant-mother interaction; therefore, the development of emotion regulation abilities in these infants and toddlers may be delayed, which explains to some extent the causes of the delayed development of social smiling and active social engagement in children with ASD.

From the results of the correlation analysis, there was a difference between age and the developmental level and social behaviors of some infants and toddlers in the HR group and the TD group, but the difference was not representative, i.e., the age and developmental level of the infants and toddlers did not influence their social behaviors under general conditions. For infants and toddlers with HR, the analysis of the correlation between clinical symptoms and social behaviors showed that there was no correlation between social behaviors and symptom severity during the FF episode of the SFP. During the SF episode, the duration of eye contact by infants and toddlers with HR was positively correlated with the symbolic behavior factor score and the total CSBS-DP score; the duration of social smiling was positively correlated with the social communication factor score and the total CSBS-DP score; and the duration of active social engagement was positively correlated with the social communication factor score, the symbolic behavior factor score and the total CSBS-DP score; and the frequency of eye contact was positively correlated with the social communication factor score, the language factor score and the total CSBS-DP score. The results indicated that the more flexible and appropriate the eye contact and active social engagement of the infants and toddlers with HR, the less severe were the ASD symptoms, which is also consistent with the results of most studies (39–41). Although the social behaviors of infants and toddlers with ASD develop over time, their development level is limited, the gap between infants and toddlers with ASD and infants and toddlers with TD also increases over time, and infants and toddlers with ASD develop more clinical symptoms of ASD.

The SFP presents changes in children's expressions, emotions and behaviors in a more microscopic coding mode. On the basis of setting a normal interaction, the SFP provides a social challenge scenario by setting the SF episode, during which the social signals of the mother are completely missing during the period. For typically developing children, their inability to adapt to the loss of social signals stimulated their ability to initiate social interactions, express emotions, regulate emotions, and bear stress. The analysis of the above results showed that the social behaviors in infants and toddlers with HR, especially their social behaviors during the SF episode of the SFP, were associated with the core ASD symptoms. According to the extreme male brain theory of autism (42, 43) the toddlers with ASD are more prone to over systematization and thus have lower empathic ability than do TD toddlers, making them more prone to deficiencies in social and verbal communication. By examining the differences in social behaviors between the infants and toddlers in the HR group and the TD group, we found that although there were many differences in the abnormal social behaviors between infants and toddlers with ASD and infants and toddlers with TD during the FF and SF episodes of the SFP, the social behaviors of infants and toddlers with HR, such as eye contact and active social engagement, during the SF episode (a frustration scenario), were significantly correlated with core communication impairments, such as the social communication factor score, the symbolic behavior factor score and the total CSBS-DP score. That is, the SF episode of the SFP can better induce the social communication impairments in infants and toddlers with ASD. Markram et al. (44) proposed the intense world theory, suggesting that an excessively active brain would excessively amplify ordinary sensory experiences, causing the toddlers with ASD to be in a state of fragmented sensory information and to be overloaded, and because of such a strong reaction, the intense emotions perceived by them from the surrounding environment causes social withdrawal, resulting in a series of autism symptoms such as social communication impairments and stereotyped behaviors. Therefore, facing social communication challenges such as the SF episode, infants and toddlers with TD attempted to arouse their mothers' responses by pointing and social smiling. In contrast, for infants and toddlers with HR, even for those who had higher function, they may have had good interactions with their mothers during the FF episode, but when their mothers did not respond, social pressure was reduced. They made fewer attempts or shorter attempts to initiate social interactions.

In addition, some studies have shown that when responding to emotional reactions, children with ASD have worse emotion regulation abilities and more unreasonable expression and are more likely to show negative emotions (45). However, in this study, the negative emotions (protest behavior and non-social smiling) in infants and toddlers with HR and TD were not different, and there were no differences during the frustration scenario, i.e., when the mothers used still faces. Further validation and discussion are needed in future studies.

Based on the re-diagnosis and regrouping of the children at 2 years of age, machine learning methods, including SVM, naïve Bayes and random forest, were used to construct models for early ASD screening. And we found the classification model established using the SVM had the best performance, especially it was found to have better screening ability and reliability for the SF episode. Unfortunately, when the model was selected and applied to the differential diagnosis of the children in the HR group, the 5 N-ASD was not classified correctly. And also, it is the goal to identify N-ASD from ASD group in our future efforts. Since the age differences between the ASD group and TD group, we added the Figure S2, in which we divided ASD group and TD group (total 83 kids) into 4 groups with month age, respectively, 8–11, 12–15, 16–19, and 20–23, and we found there were not regularity between the classification accuracy vs. month age.

Similarly, there are limitations in this study. First, because infants and toddlers with ASD generally have delayed development, the 2 groups were not matched by age to make the development level of the HR group the same as that of the TD group. Second, the sample size was small. In view of these limitations, we will continue to expand the sample size in future studies to further verify the findings under controlled physiological and psychological ages. We hope that the SFP will be widely applied for the early ASD screening and that a more objective, standardized and convenient way for self-screening at home will be achieved.

## Data Availability Statement

All datasets generated for this study are included in the article/Supplementary Material.

## Ethics Statement

The studies involving human participants were reviewed and approved by the Medical Ethics Committee of Nanjing Brain Hospital Affiliated to Nanjing Medical University. Written informed consent to participate in this study was provided by the participants' legal guardian/next of kin. Written informed consent was obtained from the individual(s), and minor(s)' legal guardian/next of kin, for the publication of any potentially identifiable images or data included in this article.

## Author Contributions

NQ and XK designed experiments. NQ, CT, MZ, JW, CL, XX, JF, LZ, TX, and HF carried out experiments. NQ, CT, and WH analyzed experimental results. NQ wrote the manuscript.

## Conflict of Interest

The authors declare that the research was conducted in the absence of any commercial or financial relationships that could be construed as a potential conflict of interest.
