# Supplementary material for: Application of the Still-Face Paradigm in Early Screening for High-Risk Autism Spectrum Disorder in Infants and Toddlers
Source: Front Pediatr. 2020 Jun 5;8:290. doi: 10.3389/fped.2020.00290 (PMC7290044; doi:10.3389/fped.2020.00290)
Supplement: Supplementary file 1 [file Data_Sheet_1.docx]

**Supplemental Materials**

**
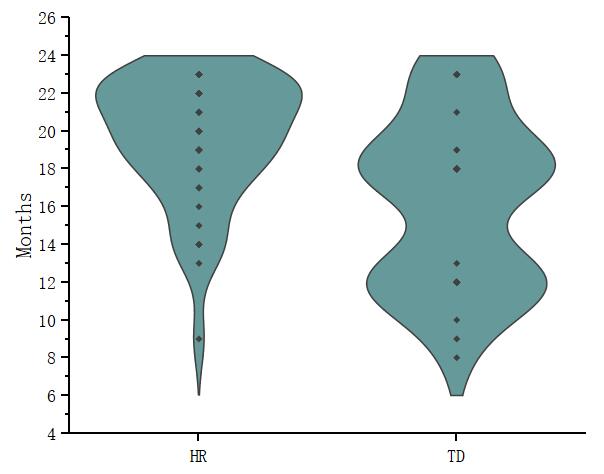
Figure S1 Comparison of the age between the HR group and the TD group**

HR, high-risk autism spectrum disorder; TD, typical development

**
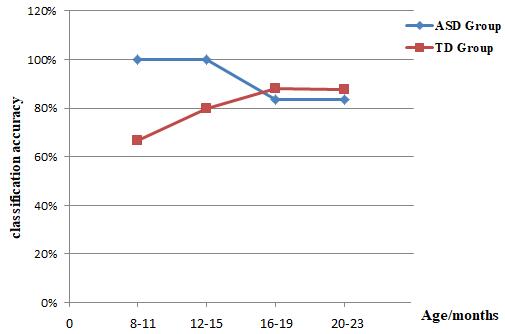

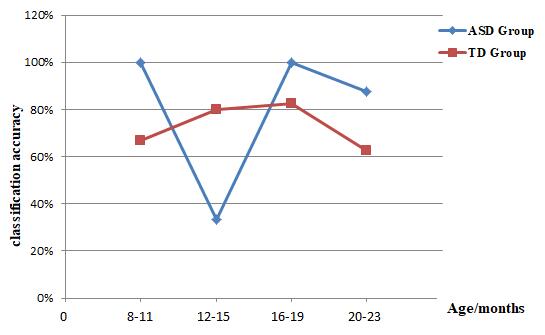
Figure S2 the relationship of the classification accuracy and age between ASD group and TD group**

Figure S2b the relationship of the classification accuracy and age between ASD group and TD group in SF episode

Figure S2a the relationship of the classification accuracy and age between ASD group and TD group in FF episode

ASD, autism spectrum disorder; TD, typical development
